# Cytokines Involved in the Pathogenesis of SSc and Problems in the Development of Anti-Cytokine Therapy

**DOI:** 10.3390/cells10051104

**Published:** 2021-05-04

**Authors:** Yoshihito Shima

**Affiliations:** Laboratory of Thermo-therapeutics for Vascular Dysfunction, Osaka University Graduate School of Medicine, 3-1 Yamada-oka, Suita, Osaka 567-0871, Japan; ryanjin@imed3.med.osaka-u.ac.jp; Tel.: +81-6-6105-5710

**Keywords:** systemic sclerosis, interleukin-6, interleukin-13, tocilizumab

## Abstract

Systemic sclerosis (SSc) is a connective tissue disease of unknown etiology. SSc causes damage to the skin and various organs including the lungs, heart, and digestive tract, but the extent of the damage varies from patient to patient. The pathology of SSc includes ischemia, inflammation, and fibrosis, but the degree of progression varies from case to case. Many cytokines have been reported to be involved in the pathogenesis of SSc: interleukin-6 is associated with inflammation and transforming growth factor-β and interleukin-13 are associated with fibrosis. Therapeutic methods to control these cytokines have been proposed; however, which cytokines have a dominant role in SSc might differ depending on the extent of visceral lesions and the stage of disease progression. Therefore, it is necessary to consider the disease state of the patient to be targeted and the type of evaluation method when an anti-cytokine therapy is conducted. Here, we review the pathology of SSc and potential cytokine targets, especially interleukin-6, as well as the use of anti-cytokine therapy for SSc.

## 1. Introduction

Systemic sclerosis (SSc) is a connective tissue disease characterized by abnormal peripheral vessels and fibrotic changes in the skin and visceral organs. Two types of SSc can be distinguished by the spread of the sclerotic skin area. Systemic skin involvement is referred to as diffuse cutaneous SSc (dcSSc) and sclerosis limited to the fingers, hands, and forearms is known as limited cutaneous SSc (lcSSc) [1]. Both types exhibit fibrotic changes in visceral organs, especially the esophagus, lungs, and heart. Most patients with dcSSc are positive for the anti-topoisomerase-1 antibody (anti-Scl-70 antibody) or the anti-RNA polymerase III antibody, and most lcSSc patients have an anti-centromere antibody present in their sera [2,3]. The clinical symptoms of SSc include vascular damage, inflammation, and fibrosis. The etiology of SSc has not been identified, but many cytokines and chemokines associated with its pathological state have been reported. Here, we explain the clinical and pathological features of SSc and introduce these cytokines, with a focus on the proinflammatory cytokine interleukin (IL)-6 and the fibrosis-related cytokines IL-13 and transforming growth factor (TGF)-β.

## 2. Clinical Stages of SSc

In most cases of SSc, the first symptom is Raynaud’s phenomenon [4]. Upon receiving a cold stimulus, the color of a patient’s fingers changes to white, purple, and then red, which is thought to occur as a result of the cold stimulus causing blood vessel spasms, resulting in ischemia. Capillaroscopy analysis (using a microscope to observe capillaries) of patients with SSc demonstrated deformed and reduced numbers of capillaries [5]. Normal capillaries in the nail fold are hairpin-shaped. In the early stages of SSc, the tip of the hairpin bulges to form a “giant loop” and blood leaks from the hairpin tip to form “nail fold bleeding points.” As the disease stage progresses, the capillaries show meandering, branching, and abnormal anastomosis. In the late stages, the density of capillaries decreases. Because the supply of blood flow to the fingertips is insufficient, fingertip ulcers or fingerpad atrophy can occur.

After Raynaud’s phenomenon, the fingers become swollen and edematous, followed by a gradual hardening of the skin. This causes limitations on the range of joint motions leading to reduced daily activities [6,7]. Tissue fibrosis is also observed in the lungs. When the alveolar septal wall becomes fibrotic and thickened, respiration becomes difficult leading to a restrictive lung disorder. In addition, thickening of the alveolar septal wall reduces gas diffusibility which leads to a decreased percentage of vital capacity (%VC) and percentage of diffusing capacity for carbon monoxide (%DLco) determined by respiratory function tests [8,9]. Chest computed tomography images from SSc patients indicated elevated density in the lung below the pleural area on the dorsal side of the lower lung field and traction bronchial ectasia due to lung tissue contraction.

Healthy esophageal tissue consists of the mucosa, a circular muscular layer, a longitudinal muscle layer, and the adventitia [10]. When hardening and atrophy of the muscular layer occur, flexible movement of the esophagus becomes impaired [11]. Additionally, the esophageal diameter is expanded because of a decrease in the contractile force due to fibrosis of the muscular layer. Furthermore, when the diameter of the esophagus is expanded, the long axis diameter of the esophagus is shortened, resulting in an esophageal hiatal hernia [12]. Healthy lower esophageal sphincter muscles contract normally to prevent backflow of the contents of the stomach and relax after swallowing to discharge swallowed objects into the stomach [10]. Sclerosis of the lower esophageal sphincter makes it difficult to discharge the swallowed food into the stomach, and the stomach contents easily flow back into the esophagus [13]. In these cases, the esophageal mucosa is constantly exposed to strong acids due to the backflow of gastric juices, and structural reflux esophagitis occurs [14]. Fibrotic changes in the lower intestinal tract reduce the digestion and absorption of food and nutrients. Therefore, patients with SSc often have poor nutritional status [15]. In addition, movement disturbances in the lower digestive tract cause bowel movements to be stagnant and some patients with SSc develop pseudo intestinal obstruction if the intestinal tract occlusion is due to a movement disorder of the gastrointestinal tract.

## 3. Progression of Pathological Stages

SSc, especially dcSSc, is considered to progress stepwise from the onset of the disease. The frequency of the occurrence of visceral lesions of SSc varies depending on the duration of the disease [16]. We propose dividing the clinical course of SSc into four stages as follows: vascular damage and ischemia, inflammation, tissue fibrosis, and tissue atrophy.

The earliest stage involves vascular damage followed by ischemic changes. Raynaud’s phenomenon is an early event of SSc [17]. Pathological studies revealed the infiltration of mononuclear cells into the perivascular area before sclerosis of the skin occurs [18,19]. The next stage involves tissue inflammation and edema. Histopathologically, edema of the papillary and reticular dermis was seen in tissues at this stage. Swelling was observed in the patient’s fingers, the dorsal surface of the hands, and forearms [18]. Fibrotic changes and tissue sclerosis may be induced in the next stage leading to the restricted movement of the fingers, wrist, and mouth. Finger dexterity is impaired. In addition, the extracellular matrix volume increases, and collagen fibers become thick and tight in the subcutaneous layer of the skin, making the patient’s skin difficult to pinch [20]. The invasion of organs differs depending on the patient, but in many cases, tissue sclerosis is observed in the esophagus, lungs, or lower intestinal tract [16]. As the fibrosis progresses, the fat layer disappears from the subcutaneous tissue, and eventually, adhesion between the skin and the fascia occurs due to the atrophy of the subcutaneous tissue itself. The fingers and arms become thin due to the decreased amount of subcutaneous tissue. The final stage of SSc is tissue atrophy [20] where the fingers show foreshortening because of atrophic changes and the face exhibits a shiny masked appearance.

## 4. Cytokines Involved in the Pathology of SSc

As mentioned above, SSc has various pathological features including vascular dam-age, inflammation, and fibrosis. When searching for “systemic sclerosis” and cytokines in the PubMed database, TGF-β had the highest number of hits, followed by IL-6, IL-4, tumor necrosis factor (TNF)-α, platelet-derived growth factor, IL-10, IL-13, IL-1, IL-2, and IL-17 indicating cytokines are related to the pathogenesis of SSc. Of these cytokines, IL-2, IL-4, IL-10, IL-13, and IL-17 are mainly produced by T lymphocytes, but IL-6 and TGF-β can be secreted by many types of cells, including T cells. This chapter describes the evidence for the biological and clinical roles of these cytokines in SSC, especially IL-6.

TGF-β was discovered in 1978 in the culture supernatant of retrovirus-transformed fibroblasts as a factor that transforms normal rat renal fibroblasts [21]. TGF-β promotes the production of proteins that form the extracellular matrix including collagen and fibronectin, and is thought to be involved in fibrosis of the SSc dermis. Kulozik et al. reported in 1990 that TGF-β mRNA was expressed around blood vessels in the inflammatory phase of SSc [22]. Kawaguchi et al. reported that a culture of fibroblasts from SSc patients produced TGF-β and they also reported the production of prolyl 4-hydroxylase, an enzyme that regulates procollagen production by TGF-β, was higher in fibroblast cultures from SSc patients than from healthy subjects [23]. Yamakage et al. reported that TGF-β and platelet-derived growth factor-AA promoted fibroblast proliferation using SSc skin samples [24]. TGF-β is thought to induce fibroblast proliferation and enhance extracellular matrix production.

IL-2 was discovered as a T cell growth factor in the culture supernatants of human peripheral blood lymphocytes stimulated with phytohemagglutinin [25]. Th2 cells produce IL-4, IL-5, IL-6, IL-10, and IL-13. These cytokines produced by Th2 cells are thought to be involved in the pathogenesis of SSc as described below, and the induction of Th2 cells by IL-2 is expected to be a more upstream abnormality in the pathogenesis of SSc. Umehara et al. reported high levels of IL-2 in the culture supernatants of peripheral blood mononuclear cells isolated from patients with SSc [26], and soluble IL-2 receptor, to which IL-2 binds, was highly concentrated in the sera of SSc patients [27], which correlated with mortality. Hawrylko et al. reported that CD4 positive peripheral blood mononuclear cells from SSc patients produced IL-2 in response to type 1 collagen stimulation [28].

IL-4 was identified as a B cell stimulating factor (B cell growth factor) in the culture supernatants of thymoma cells [29]. IL-4 induces naive T cells to differentiate into Th2 cells, which produce IL-4 and express IL-4 receptors, suggesting a positive feedback mechanism. IL-4 is present in bronchial asthma and atopic dermatitis patients. High levels of IL-4 were reported in alveolar lavage fluid of bronchial asthma, and IL-4 induced high [^3^H]-tritium uptake when incubated with peripheral blood mononuclear cells from atopic dermatitis patients. This is because IL-4 acts on the B-cell lineage to induce IgE production and on basophils and mast cells to increase the expression of IgE receptors [30]. The addition of IL-4 to human fibroblasts increased type 1 and 3 procollagens, and fibronectin mRNAs [31]. Famularo et al. reported that IL-4 concentrations were high in the culture supernatants of SSc patient sera and patient peripheral blood mononuclear cells [32]. These reports suggest a link between IL-4 and extracellular matrix production. Hasegawa et al. similarly reported high IL-4 levels in SSc patient sera [33]. Tenascin, an extracellular matrix factor involved in wound healing, is elevated in fibrotic diseases such as SSc, and its expression in skin fibroblasts is strongly induced by IL-4. High levels of tenascin and IL-4 were reported in the skin lesions in SSc patients [34], and tenascin production from human fibroblasts was enhanced by IL-4 [35].

IL-13 shares its α chain receptor with the IL-4 receptor and is thought to be functionally redundant with IL-4 [36]. In addition to IL-4, IL-13 has an important role in allergic diseases such as bronchial asthma. According to a report by Hasegawa et al., serum from SSc patients had high levels of IL-13 and IL-4 [33]. IL-13 is secreted by Th2 cells and induces fibroblasts to become myofibroblasts, which in turn induces the formation of the extracellular matrix and tissue stiffening. Bronchial wall remodeling occurs in bronchial asthma with prolonged disease duration and IL-13 acts with IL-4 to induce fibrosis after repeated inflammation [37]. Although the main source of IL-13 is generally thought to be CD4-positive Th2 cells, Fuschiotti et al. reported CD8-positive cells in the peripheral blood of SSc patients produced high levels of IL-13, which was more pronounced in dcSSc patients who develop extensive skin sclerosis compared with lcSSc patients, where skin sclerosis is limited [38]. These findings suggest IL-13 is involved in the fibrotic sclerosis of tissues. IL-13 is also produced by innate lymphoid cells (ILC2) [39], and the number of ILC2 cells in the skin and blood of SSc patients was increased compared with healthy individuals, which correlated with the skin score and the degree of pulmonary fibrosis [40].

IL-10, a cytokine synthesis inhibitory factor [41], is produced by various cells including T cells and acts mainly on Th1 cells to suppress their cytokine production [42]. Hasegawa et al. reported that IL-10 levels were higher in the sera of SSc patients than in the control group [33]. Because inflammatory bowel disease develops in IL-10 knockout mice, IL-10 is thought to have an inhibitory effect on inflammation [43]. Indeed, the elevation of IL-10 observed in SSc patients may be a feedback loop to increase inflammatory cytokines such as IL-6.

IL-17A is a proinflammatory cytokine produced by Th17 cells or natural killer cells [44]. IL-17A enhances neutrophil migration and activation, stimulates the production of nitric oxide and IL-6, and increases the expression of cyclooxygenase-2, an enzyme in the arachidonic acid cascade that forms prostaglandin I2 and E2. Because prostaglandins I2 and E2, as well as nitric oxide are vasodilators, IL-17A is thought to induce neutrophil migration through dilated vascular walls. Kurasawa et al. reported that IL-17 mRNA expression was increased in the lesional skin and peripheral blood mononuclear cells of SSc patients, and IL-17 levels were high in their serum [45]. Of note, IL-17 levels were high in patients with severe lung lesions [46].

**Figure 1:**
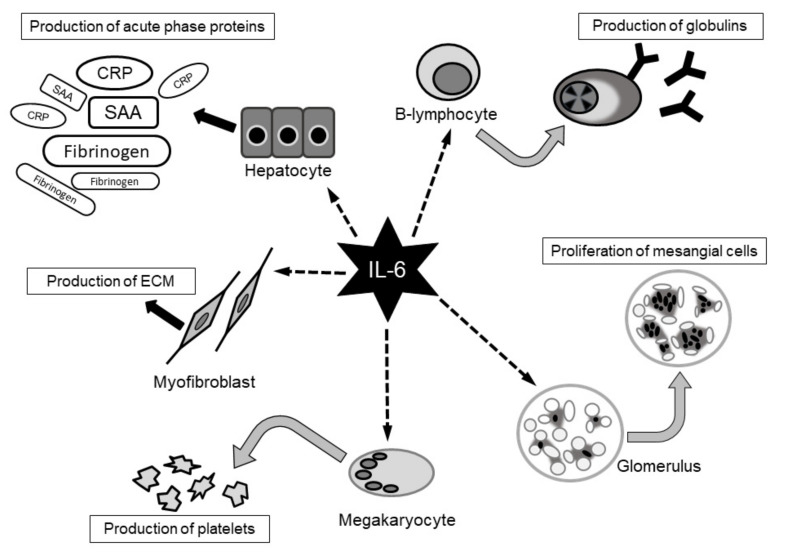
Pleiotropic functions of interleukin-6. It has been reported interluukin-6(IL-6) has various effect [49]. IL-6: interleukin-6, CRP: C-reactive protein, SAA: serum amyloid A, ECM: extracellular matrix.

IL-6 was originally identified as a B-lymphocyte stimulating factor by Kishimoto et al. in 1983, and its cDNA was cloned by Hirano et al. in 1986 [47,48]. In the 1980s, several new molecules were found to be involved in inflammation and plasmacytoma growth, which were referred to as interferon-β2, hepatocyte stimulatory factor, and hybridoma plasmacytoma growth factor. Subsequently, these were revealed to be the same molecule and were renamed IL-6. As the variety of names show, IL-6 has numerous biological functions. Immunocompetent cells, neural cells, and mesangial cells are all affected by stimulation with IL-6. IL-6 stimulates hepatocytes, resulting in the elevation of C-reactive protein (CRP), fibrinogen, and serum amyloid A protein. This hepatic activation explains why patients in an inflammatory state show elevated CRP levels and blood sedimentation rates. IL-6 is also involved in the pathogenesis of rheumatoid arthritis. IL-6 is synthesized locally in the joints, producing redness and swelling, where it binds to IL-6 receptors expressed by hepatocytes to induce the production of CRP, serum amyloid A protein, and fibrinogen [49] (Figure 1).

**Figure 2:**
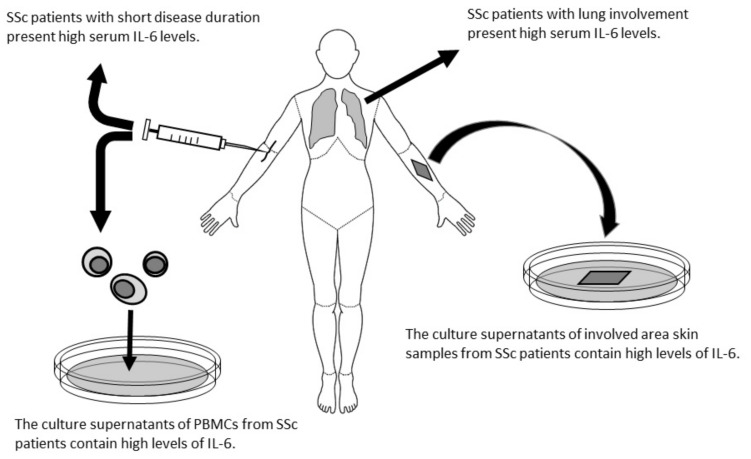
Schematic explanation about the reports that IL-6 is involved in the pathogenesis of SSc [50,51,52,53,54]. IL-6: interleukin-6, PBMCs: peripheral mononuclear cells.

Taken together, IL-6 is considered to have an important role in the pathogenesis of SSc. Needleman et al. reported measurable IL-6 levels in the sera of SSc patients using a bioassay method because there was no enzyme-linked immunosorbent assay system for IL-6 [50]. Feghali et al. reported primary cultures of skin tissue isolated from SSc patients had high concentrations of IL-6 [51]. Furthermore, culture supernatants of skin fibroblasts isolated from an area of affected skin in a patient with SSc contained a 6- to 30-fold greater concentration of IL-6 compared with unaffected skin or control skin fibroblast supernatants. Their report suggests that IL-6 may be produced in the affected lesion. In contrast, Gurram et al. reported higher IL-6 production from peripheral mononuclear cells isolated from SSc patients than from normal mononuclear cells [52]. The supernatants of peripheral blood mononuclear cells from SSc patients cultured with type I collagen contained higher amounts of IL-6 compared with normal control cultures. Sato et al. analyzed thirteen cytokines and chemokines in sera from SSc patients and found that IL-6 levels correlated with modified Rodnan skin scores (mRSS) that represent the degree of skin involvement [53] (Figure 2).

## 5. Problems with Pathogenic Cytokines

**Figure 3:**
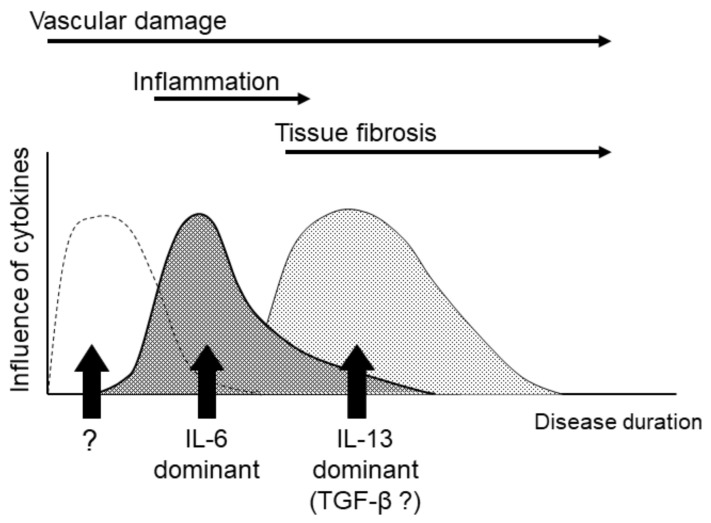
The hypothesis of cytokine alterations. Cytokines that mainly act on the pathogenesis of SSc may be replaced as the disease progresses. IL-6: interleukin-6, IL-13: interleukin-13, TGF-β: transforming growth factor-β.

As described above, many cytokines have been reported to be involved in the pathogenesis of SSc. However, SSc, and especially dcSSc, undergoes several pathological changes from the early to late stages of the disease (damaged capillaries, inflammatory edema, fibrosis, and atrophy) and different cytokines might be involved in each of these stages. Hasegawa et al. analyzed serum IL-6 levels following the categorization of samples into early phase (<3 years) dcSSc, early phase lcSSc, late phase (over 3 years) dcSSc, and late phase lcSSc. They found that the serum samples from early phase dcSSc patients contained higher levels of IL-6 than the other groups [55]. This report suggests that IL-6 might have a role in the pathogenesis of SSc, but possibly only during a specific stage or state of SSc. Matsushita et al. reported that serum levels of IL-6 and IL-10 at initial diagnosis were significantly higher in SSc patients than in controls, but that both cytokines were decreased at 6 years after the initial diagnosis. In contrast to IL-6 and IL-10, serum levels of IL-12 in the early stages of the disease were significantly lower than those in the control group but were increased 15.3 times within 6 years after the initial diagnosis [56]. We reported that the patients who showed a higher level of serum IL-6 among the patients surveyed were relatively short disease durations and serum IL-6 levels are inversely proportional to serum IL-13 levels [57]. That is, the patients with higher serum IL-13 presented lower IL-6. Thus, it is likely that the cytokines involved in the pathogenesis of SSc switch depending on the length of the disease history. In rheumatoid arthritis, the effects of TNF-α and IL-6 are observed regardless of the length of the disease, but in SSc, IL-6 may have a minimal role in patients with a long disease course and IL-13 may have a limited role during the early disease stages. (Figure 3) As mentioned above, IL-6 is a proinflammatory cytokine, but recently, it was reported that the phosphodiesterase type 5 inhibitor (PDE5i) sildenafil was effective for Raynaud’s phenomenon by suppressing the production of IL-6 from dermal fibroblasts [58]. Therefore, IL-6 might act at the stage of vascular damage.

A further complication factor is that cytokines which work dominantly in the pathogenesis may be different depending on the affected organs. Many studies have reported a relationship between IL-6 and the involvement of internal organs, especially lung fibrosis. Crestani et al. cultured alveolar macrophages from bronchoalveolar lavage collected from eleven lung fibrosis patients complicated with SSc and eight normal subjects [59]. IL-6 levels in the SSc culture supernatants were higher than in normal supernatants although there was little difference in response to lipopolysaccharide stimulation. An inverse correlation between serum IL-6 levels and the %VC in the early stage dcSSc group was also reported [60]. Scala et al. measured IL-6 concentrations in sera from twenty SSc patients and found that samples from dcSSc with lung involvement contained higher levels of IL-6 [54]. These reports suggest the possible involvement of IL-6 in lung fibrosis, which is an important symptom frequently observed in SSc patients.

## 6. Anti-Cytokine Therapy

Various anti-cytokine therapies with biologics have been investigated. dcSSc patients were treated with CAT-192, a neutralizing anti-TGF-β antibody [61] at three different dos-es: 0.5, 5, and 10 mg/kg. After 6 months of observation, no significant decrease in mRSS was observed in the active treatment group although the mRSS tended to be decreased in patients with longer disease duration.

Anti-IL-6 therapies have also been examined. Tocilizumab (TCZ), a monoclonal antibody against human IL-6R, was developed by Chugai Pharmaceutical Co., Ltd. in Japan, and is now used as a therapy for Castleman disease, Takayasu arteritis, giant cell arteritis, idiopathic juvenile arthritis, and rheumatoid arthritis. TCZ inhibits the binding of IL-6 to IL-6R. Cells expressing IL-6R are limited to hepatocytes, but the signal transduction molecule gp130 is expressed in most human cells [62]. The soluble form of IL-6R is present in serum and can generate an inflammatory signal via gp130 when it binds to IL-6. Therefore, inhibiting the action of IL-6R is a reasonable strategy to block IL-6 function. We reported that SSc cases treated with TCZ had decreased mRSS and the skin hardness evaluated by Vesmeter, a device that measures the hardness, elasticity, and viscosity of skin [63]. On the basis of these findings, 87 patients with SSc were administered TCZ or placebo to examine the therapeutic effect of TCZ in a phase II randomized controlled trial (faSScinate study) in 2012 [64]. The least-squares change of mRSS at 24 weeks after administration was −3.92 in the TCZ group and −1.22 in the placebo group. At 48 weeks, the least-squares change of mRSS was −6.33 in the TCZ group and −2.77 in the placebo group. The TCZ patients showed a tendency toward a greater reduction of mRSS, and they also showed smaller reductions of forced vital lung capacity than those of the placebo group. To verify these results, a new expanded multicenter trial in 27 countries (focuSSced study) had conducted. It was expected that the decrease in mRSS would become clear, but the variability of change in mRSS was getting widely, and the results seen in the faSScinate study have been blurred [65]. In the focuSSced study, the entry criteria were patients within 60 months of onset, based on the understanding that the inflammation stage of SSc is early in the disease course. Although the decreased skin scores in the TCZ group did not reach statistical significance compared with the placebo group, the pulmonary function test demonstrated that the average of the decrease of forced vital lung capacity in the TCZ group was smaller than that of the placebo group. TCZ is now approved by the United States Food and Drug Administration to treat lung involvement in SSc patients.

Recently, a clinical trial of romilkimab, a bispecific immunoglobulin G4 that neutralizes IL-4 and IL-13, demonstrated changes in the mRSS of dcSSc patients. At 24 weeks of observation, the romilkimab group had a significantly lower mRSS than the placebo group [66]. As described above, anti-IL-6 therapy may be successful in patients with a short disease duration and anti-IL-13 therapy might be successful in patients with advanced-stage disease.

## 7. Evaluation of Anti-Cytokine Therapy

It is expected that various anti-cytokine therapies will be approved in the future, but there are many problems to overcome before their practical use. For SSc, it will be necessary to decide the target stage and the target involved organs. In the focuSSced study, the entry criteria were patients within 60 months of onset, based on the understanding that the inflammation stage of SSc is early in the disease. A disadvantage of large-scale trials targeting SSc is that the condition of SSc patients differs depending on their disease stage. IL-6 and TNF-α have important roles in the early- and late-stage pathogenesis of rheumatoid arthritis. However, factors involved in the pathogenesis of SSc are thought to change depending on the stage of disease progression. Furthermore, the speed of disease progression caries in each patient. We proposed a hypothesis that TCZ has variable efficacy in SSc patients. To investigate our hypothesis, we divided fifteen SSc patients into two groups, an add-on TCZ group and a group where only conventional treatment was continued. We followed the changes of mRSS for 6 months and then compared these results with baseline cytokines, chemokines, adhesion molecules, or sIL-6R. As we hypothesized, the degree of mRSS reduction in the TCZ added-on group correlated with a shorter disease duration and higher CRP values. As described above, patients with high serum IL-13 levels did not respond to TCZ treatment [57]. SSc patients with high levels of serum IL-13 may be in the fibrotic stage and are considered to have lost reactivity to TCZ.

Another problem is how to evaluate the effect of anti-cytokine treatment. The historical judgement that mRSS must be used as the main evaluation criterion renders the development of therapeutic drugs for SSc treatment difficult. From the results of the focuSSced study, it is obvious that it is not appropriate to use mRSS as an evaluation meth-od for large-scale studies. In large-scale clinical trials, the mRSS value should be closer to the true value; however, there will also be a greater variation in the mRSS. We used a Vesmeter [67] and several other devices have been proposed to evaluate the skin involvement of SSc including the Durometer [68] and Cutometer [69]. If the most appropriate target of anti-cytokine drugs is the skin, studies should use such a device. As a result, spirometry appears to be the most suitable evaluation method for TCZ. Because factors involved in the pathogenesis of SSc are considered to differ depending on the disease stage and affected organs, it is important to use an effective evaluation method.

## Data Availability

No new data were created or analyzed in this study. Data sharing is not applicable to this article.
